# Biochemical Features of a Catalytic Antibody Light Chain, 22F6, Prepared from Human Lymphocytes*[Fn FN2]

**DOI:** 10.1074/jbc.M113.454579

**Published:** 2013-05-15

**Authors:** Emi Hifumi, Naoko Fujimoto, Mitsue Arakawa, Eri Saito, Shingo Matsumoto, Nobuyuki Kobayashi, Taizo Uda

**Affiliations:** From the ‡ Research Center for Applied Medical Engineering, Oita University, Dan-noharu 700, Oita-shi, Oita 870-1192, Japan,; the ¶ Department of Applied Chemistry, Faculty of Engineering, Oita University, 700 Dan-no-haru, Oita 870-1192, Japan,; the ‖ Department of Nursing, Faculty of Medicine, Oita University, 1-1 Idaigaoka Hazamamachi, Yufu-city, Oita 879-5593, Japan,; the ** School of Pharmaceutical Sciences, Nagasaki University, 1-14 Bunkyo-cho, Nagasaki-shi, Nagasaki 852-8521, Japan, and; § JST-CREST (Japan Science and Technology Corporation), Kawaguchi-city 332-0012, Japan

**Keywords:** DNase, Enzyme Catalysis, Influenza Virus, Molecular Modeling, RNA Catalysis, Catalytic Antibody, Human Light Chain, Influenza Virus Type A

## Abstract

Human antibody light chains belonging to subgroup II of germ line genes were amplified by a seminested PCR technique using B-lymphocytes taken from a human adult infected with influenza virus. Each gene of the human light chains was transferred into the *Escherichia coli* system. The recovered light chain was highly purified using a two-step purification system. Light chain 22F6 showed interesting catalytic features. The light chain cleaved a peptide bond of synthetic peptidyl-4-methyl-coumaryl-7-amide (MCA) substrates, such as QAR-MCA and EAR-MCA, indicating amidase activity. It also hydrolyzed a phosphodiester bond of both DNA and RNA. From the analysis of amino acid sequences and molecular modeling, the 22F6 light chain possesses two kinds of active sites as amidase and nuclease in close distances. The 22F6 catalytic light chain could suppress the infection of influenza virus type A (H1N1) of Madin-Darby canine kidney cells in an *in vitro* assay. In addition, the catalytic light chain clearly inhibited the infection of the influenza virus of BALB/c mice via nasal administration in an *in vivo* assay. In the experiment, the titer in the serum of the mice coinfected with the 22F6 light chain and H1N1 virus became considerably lowered compared with that of 22F6-non-coinfected mice. Note that the catalytic light chain was prepared from human peripheral lymphocyte and plays an important role in preventing infection by influenza virus. Considering the fact that the human light chain did not show any acute toxicity for mice, our procedure developed in this study must be unique and noteworthy for developing new drugs.

## Teaser

**Background:** We explored catalytic antibodies applicable to patients.

**Results:** A human light chain (22F6) possessing both amidase and nuclease activities and preventing infection of influenza virus was obtained.

**Conclusion:** The 22F6 light chain holds a huge potential as a new drug for patient therapies in the future.

**Significance:** The procedure developed in this study is highly noteworthy for developing new drugs.

## Introduction

Natural type antibodies and their subunits (light and heavy chains) possessing catalytic features have been extensively studied for the past 2 decades. As a result, some unique catalytic antibodies have been successfully produced from the viewpoint of various reactivities. Catalytic antibodies, such as vasoactive intestinal peptide (1), DNA (2), HIV gp41 (3), HIV gp120 (4), and factor VIII (5), exhibited degradation abilities against antigens, as reported by Paul *et al.* (4), Gabibov and co-workers (2), Uda and co-workers (3), and Kaveri and co-workers (7). Regarding the preparation of a catalytic antibody, Paul *et al.* (4) proposed a unique method named “covalently reactive analog,” which derived a catalytic antibody against HIV (6). The physiological role with respect to autoimmunity in humans was clarified by Kaveri and co-workers (7). In the case of Ponomarenko *et al.* (8), they obtained reactive autoantibodies (from the sera of humans with multiple sclerosis) to specifically cleave myelin basic protein but not other proteins. Nevinsky and co-workers (9, 10) purified catalytic antibodies cleaving DNA and RNA from the autoimmune diseases, such as systematic lupus erythematous, multiple sclerosis, Sjogren syndrome, etc. The patients bearing autoimmune diseases frequently have nuclease-like catalytic antibodies.

Recently, a unique catalytic antibody A17 named a “reactibody” was prepared by Smirnov *et al.* (11) by employing an innovative idea and technique. It could cleave paraoxon and possesses an unusual deep cavity at the interface of V_L_ and V_H_.

An antibody light chain that is a subunit of the parent antibody exhibited interesting catalytic features as a peptidase and/or proteinase capable of cleaving vasoactive intestinal peptide (1), prothrombine (12), chemokine receptor CCR-5 (13), urease of *Helicobacter pylori* (14), etc. Today, meaningful results of *in vitro* as well as *in vivo* assays are very important to medicinal applications in the near future. The catalytic light chain by Hifumi and co-workers (13) suppressed a number of *H. pylori* infecting the stomachs of mice. They also reported the good efficacy of a mouse-type catalytic antibody heavy chain in suppressing infection of influenza virus type A in an *in vitro* assay (15). In addition, they have recently developed a human type catalytic light chain capable of increasing the survival rate of suckling mice infected with the rabies virus in an *in vivo* experiment (16).

The ultimate goal of catalytic antibody research is to develop new patient therapies that utilize the advantages offered by human catalytic antibodies. Through 2 decades of study of natural type catalytic antibodies as mentioned above, that goal is coming to fruition, because such antibodies are close to actual utilization. In this study, we prepared some antibody light chain genes taken from human lymphocytes, followed by expression of the genes in *Escherichia coli*. The human antibody light chain 22F6, of 20 other light chains, showed not only catalytic features but also a substantial effect on suppression of influenza virus infection in both *in vitro* and *in vivo* assays. The unique catalytic light chain 22F6 found in this study may open up applicable uses of the catalytic antibodies in the near future.

## MATERIALS AND METHODS

### Amplification of DNA Fragments Encoding Light Chains from Germ Line Genes of Subgroup II

We obtained 100 ml of peripheral blood from a healthy volunteer immunized by previous infections of influenza viruses. Peripheral blood lymphocytes were harvested using a Ficoll-Paque (GE Healthcare) gradient, and five vials of 1.0 × 10^7^ cells/ml were stored in liquid nitrogen. Total RNA was extracted from 3.0 × 10^7^ cells using an RNA isolation kit (Stratagene, La Jolla, CA). cDNA was synthesized by reverse transcription-PCR using a total RNA template using oligo(dT) as a primer (ThermoScript RT-PCR system; Invitrogen). DNA fragments encoding human light chains were amplified from the cDNA by PCR using four primers separately as a forward primer (5′-cacctagGATATTGTGATGACCCAG-3′) and one reverse primer (5′-ACACTCTCCCCTGTTGAAGCTCTTTGTG-3′) (Table 1) including a direct insert to the TOPO site and a start codon. The PCR occurred under the following incubation conditions: 5 min at 95 °C, 35 cycles of 15 s at 95 °C, 50 s at 54 °C for annealing, and 90 s at 72 °C for extension.

The amplified DNA fragments were separated by 3% agarose gel electrophoresis. Fragments of the expected size (660 bp) were extracted using a QIAquick gel extraction kit (Qiagen, Valencia, CA). Purified PCR products were directly ligated to pET TOPO vector (Invitrogen), and the vectors were transformed into TOP10 cells continuously using a Champion pET101 directional TOPO expression kit (Invitrogen). After propagation of the vectors in the TOP10 cells, the inserted pET21 (b+) vector was repurified and transformed into BL21 Star (DE3) for expression of light chains belonging to subgroup II.

### Sequencing and Molecular Modeling

The 22F6 clone was sequenced with the ABI automated DNA sequencer model 3100 (Applied Biosystems), using the universal primer of a T7 promoter. The software GENETIX, version 8 (GENETIX, Tokyo, Japan), was used for sequence analysis and deduction of amino acid sequences. The nucleotide sequence is registered in DDBJ/EMBL/GenBank (accession number: AB723624).

Computational analyses of the antibody structures were performed using the deduced VL amino acid sequences on a work station (Octane 2; Silicon Graphics, Crafton, PA) running AbM software (Oxford Molecular, Oxford, UK) used to build models of three-dimensional molecules. The resulting Protein Data Bank data were applied to minimize the total energy by using DS-Modeling (Accelrys Software, San Diego, CA). This software uses the CHARMm algorithm for minimizing the energy of a molecule.

### Expression and Purification of Light Chain 22F6

pCR-Blunt II TOPO vectors containing DNA fragments encoding human light chains were digested by restriction enzymes NcoI and XhoI (New England BioLabs, Beverly, MA). The resulting DNA fragments were inserted between the same restriction sites of expression vector pET20b(+) (Novagen, Madison, WI). To express light chains, the expression vectors were transformed into BL21 (DE3) pLysS (Novagen). The transformant was grown at 37 °C in 1 liter of Luria-Bertani medium containing 100 μg/ml ampicillin to an *A*_600 nm_ of 0.6 and then incubated with 0.01 mm isopropyl-β-d-thiogalactopyranoside (Wako Pure Chemicals, Osaka, Japan) for 20 h at 18 °C.

Cells were harvested by centrifugation (3,500 × *g*, 4 °C, 10 min) and then resuspended in a solution (0.25 m NaCl, 25 mm Tris-HCl, pH 8.0). The cells were lysed by ultrasonication three times for 2 min each in an ice bath, followed by centrifugation (14,000 × *g*, 4 °C, 20 min). The expressed human light chain was recovered as the supernatant. A soluble fraction from *E. coli* was directly applied to nickel-nitrilotriacetic acid column chromatography (Takara, Otsu, Japan) equilibrated with 50 mm sodium phosphate, pH 7.0, containing 300 mm NaCl. The elution was performed by increasing the concentration of imidazole from 0 to 300 mm. Fractions containing 31-kDa protein (corresponding to a human light chain) in SDS-PAGE analysis were collected, dialyzed against a 50 mm sodium acetate buffer, pH 5.5, and then purified by cation exchange chromatography using a column of SP-5PW (Tosoh) with a gradient of NaCl solution (from 0.2 to 0.7 m).

By this method, a highly purified light chain (>99%) was confirmed by SDS-PAGE with silver staining. No band except for the light chain was visible to the naked eye; nor could we see any contamination of proteases coming from *E. coli*. Protein concentrations were determined by the Bradford method using a Lowry method using the DC protein assay kit (Bio-Rad).

### Western Blot Analysis

We prepared a lysed virus sample to use in the following Western blot analysis. Namely, influenza A virus (A/Hiroshima/37/2001(H1N1)) obtained by egg culture was prepared and treated with detergent (1% Triton X-100). Then the lysed virus solution was submitted to an SDS-PAGE experiment without staining. Next, the proteins were transferred from the gel onto an Immobilon-P PVDF membrane. The PVDF membrane was blocked with TBS containing 0.3% skim milk and 0.05% Tween 20. After being washed with TBS containing 0.05% Tween 20 (TBS-T), the membrane was incubated with the light chain 22F6 (50.9 μg/ml). After washing with TBS-T, goat anti-human IgG (F(ab′)_2_ labeled with horseradish peroxidase (1:250 dilution; ImmunoPure antibody, lot number JG1153174, Thermo Scientific) as a second antibody was reacted with the membrane for 1 h at room temperature (44). After four washings with TBS-T, color development was performed using True Blue POD (horseradish peroxidase) substrate (Kirkegaard & Perry Laboratories).

### Cleavage Assays

To avoid contamination in cleavage assays, most glassware, plasticware, and buffer solutions used in this experiment were sterilized by heating (180 °C, 2 h), autoclaving (121 °C, 20 min), or filtration through a 0.20-lm sterilized filter as much as possible. Most of the experiments were performed in a biological safety cabinet to avoid airborne contamination.

#### 

##### Hydrolysis of Synthetic Peptidyl Substrates (Amidase Activity)

Cleavage of the amide bond linking AMC to C-terminal amino acid in peptide-AMC substrates (Peptides International, Louisville, KY) was measured at 37 °C in PBS containing 0.05% sodium azide in 96-well plates. Purified light chains (100 μl, 4 μm) were mixed with a synthetic substrate (100 μl, 100 μm). AMC released from the substrate catalyzed by the light chains was detected by measurement of fluorescence emission at 470 nm when excited at 360 nm using a microplate reader (SpectraFluor Plus; Tecan, Maennedorf, Switzerland). The concentration of the released AMC was estimated using the fluorescence emission of 50 μm AMC as a standard value.

##### Hydrolysis of Phosphodiester Bond of Plasmid (DNase Activity)

Prior to the following nuclease activity assay, the degree of the contamination of DNase from *E. coli* etc. was investigated using an in-gel assay, which was performed in accordance with a previous study (9). As shown in supplemental Fig. S1, the results concluded that the degree of the contamination is at most about 10%, even if it is the case.

Cleavage of the phosphate bond of plasmid DNA pBR322 was investigated under an incubation time of 0–24 h with the light chain 22F6. In the solution, the final concentrations of 20 μg/ml pBR322 and 50 μg/ml light chain 22F6, 10 mm MgCl_2_, 1 mm EDTA, and 50 mm NaCl were contained in a 25 mm concentration of a Tris-HCl buffer (pH 7.5) at a reaction temperature of 37 °C. The reaction volume was 20 μl.

##### Hydrolysis of Phosphodiester Bond of RNA (RNase Activity)

Genome RNAs from influenza virus and Noda virus were prepared in accordance with past studies (17, 18). Briefly, virions were recovered from the banding fraction by ultracentrifugation. It was dissolved in a 1% Triton X-100 solution containing 10 mg/ml lysolecithin, followed by a 36–60% glycerol density gradient using ultracentrifugation. Fractions 4, 5, and 6 were recovered as highly purified RNA of the Noda virus. For the influenza virus, we failed to prepare influenza RNA because it was very unstable.

### In Vitro Assay

#### 

##### Viruses

The influenza viruses used were A/Hiroshima/37/2001 (H1N1) and A/Hiroshima/71/2001 (H3N2). These viruses were grown in MDCK^2^ cell culturing medium and harvested and stored as infectious culture fluid at −80 °C until further use.

##### Cells

MDCK cells were grown in Dulbecco's modified Eagle's medium (DMEM) supplemented with 10% fetal bovine serum.

##### Neutralization Test

Neutralization tests were performed as follows (19). The monoclonal antibody or antibody light chain diluted with phosphate-buffered saline (PBS) was mixed with an equal volume (75 μl) of influenza virus diluted with DMEM adjusted to give a final control count of about 500 pfu/0.2 ml (2,500 pfu/ml). After incubation for 24 or 48 h at 25 °C, an infectious virus titer of the mixture was calculated by a plaque assay. The antibody- or light chain-virus mixture was serially diluted in 10 steps; 0.2 ml of each mixture was inoculated into the MDCK cell monolayer, which had been seeded on a 6-well tissue culture tray (Falcon 3046; BD Biosciences). After adsorption for 60 min at 34 °C, the inocula in each well were removed and washed with PBS. The MDCK cells were covered with the first overlay DMEM containing 1.0% agarose ME (Iwai Chemical Industries, Tokyo, Japan) and 2 mg/ml acetyl trypsin (Sigma), and the trays were incubated for 2 days in a humidified 5% CO_2_ incubator at 34 °C. After the incubation, the cells were covered with the second overlay DMEM (0.005% neutral red in the first overlay medium). The plaques were counted on the following day.

### In Vivo Assay

After acclimatization of mice (BALB/cN Sea mice, 6 weeks old, female; Kyudou Co. Ltd., Kumamoto, Japan) for 1–2 weeks, 25 μl (500 pfu/mouse) of a solution including influenza virus H1N1 (A/Puerto Rico/8/34; PR-8 strain) with or without the catalytic light chain 22F6 was given by nasal administration into the mice. The mice were carefully examined for 3 weeks (21 days). From 0 to 21 days postinoculation (dpi), the body weight of each mouse was measured, and the death or survival of the mouse was also counted. At 7 and 14 dpi, photographs of the mice were taken.

### Assay of Toxicity of Human Light Chains

Acute toxicity tests were performed by AVSS Co. Ltd. (Nagasaki, Japan). Briefly, BALB/cN Sea female mice (6 weeks old) purchased from Kyudou Co. Ltd. were also employed in this experiment. Human light chains were prepared at concentrations of 1.66 and 9.67 mg/ml in PBS.

#### 

##### Single-dose Administration

After acclimatization of the mice for 1–2 weeks, three kinds of administration were done: oral, intraperitoneal, and intravenous. For the oral administration, 0.38–0.41 ml (33.2 mg/kg) was given to each mouse (*n* = 3 mice for each group) using a sonde with a 1-ml syringe (model number 110515F; Terumo Co. Ltd., Tokyo, Japan). For the intraperitoneal administration, 0.59–0.63 ml (49.8 mg/kg) was inoculated into mice (*n* = 6) using a needle (26 gauge × ½, Code NN-2613S, catalog no. 111021D; Terumo) with the 1-ml syringe used above. For the intravenous administration, 0.30–0.32 ml (8.3, 24.9, or 145.5 mg/kg) was injected into mice (*n* = 3) using a needle with a 1-ml syringe (26 gauge, catalogue no. ED-124S; NIPRO, Osaka, Japan).

##### Multiple-dose Administration

Multiple doses were intravenously administered into the BALB/cN Sea female mice (*n* = 3) every 7 days using a needle (26 gauge × ½, code NN-2613S, catalog no. 111021D; Terumo) with a syringe (catalog no. 110515F; Terumo Co. Ltd.). In this case, 0.10–0.11 ml (8.3 mg/kg; inoculation volume = 5 ml/kg, 1.66 mg/ml) was injected into the mice.

### Ethical Committee

This study was performed with the approval of the ethics committees of Oita University and Nagasaki University.

### Statistical Analysis

The statistical significance of differences between experimental groups was determined as follows. For data about the body weight and organ weight of assayed mice, an F-test was performed. When the result was equal variance, Student's *t* test was performed. For non-equal variance, an Aspin-Welch *t* test was employed to evaluate a significant difference between the control group and the group treated with the catalytic light chain. Values of *p* < 0.05 were considered significant.

## RESULTS

### Expression and Purification

**FIGURE 1.**
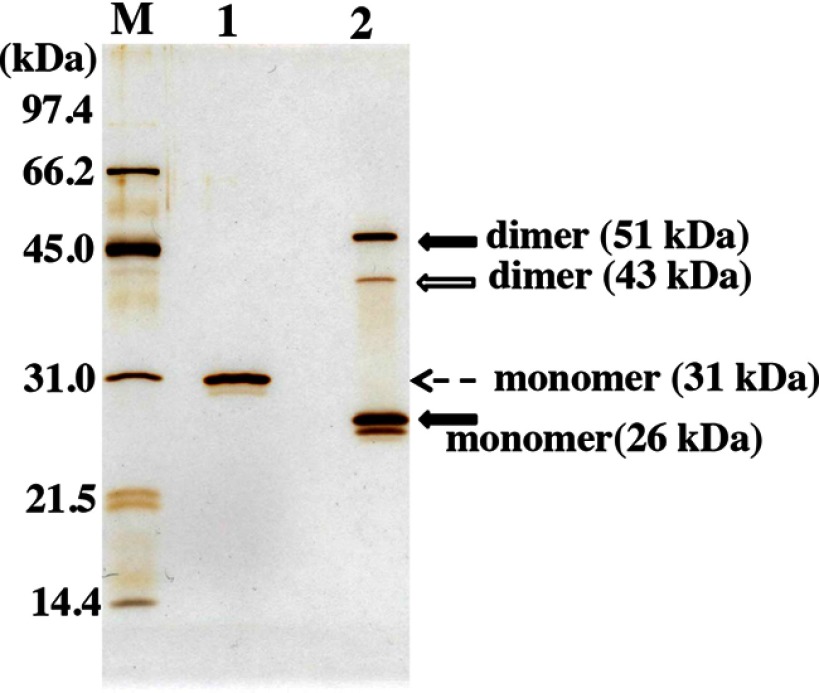
**SDS-PAGE analysis of purified light chain.** *M*, marker; *1*, reduced condition; *2*, non-reduced condition. The purified light chain sample (250 ng) was separated by 12% acrylamide gel electrophoresis and visualized by silver staining. Under the non-reduced condition, the monomeric (26-kDa) and dimeric (51- and 43-kDa) forms of 22F6 were observed. A weak band at 43 kDa may be a different dimeric form from that of the band at 51 kDa. Under the reduced condition, no other bands except for the 31 kDa band of the monomeric form were detected, indicating high purity.

Fig. 1 shows the SDS-PAGE analysis (silver staining) of the purified light chains under reduced and non-reduced conditions. The 51 and 26 kDa bands under the non-reduced condition correspond to the light chain dimer and monomer, respectively. The 43 kDa band is a dimer possessing a different surface charge from that of 51-kDa dimer. Under the reduced condition, only the 31 kDa band corresponding to the monomeric light chain was observed, and no other bands were detected. The 51 and 26 kDa bands and the 31 kDa band were confirmed as a human antibody light chain by Western blot analysis (data not shown). The light chain underwent N-terminal amino acid sequence analysis. The sequence was identified as MDIVM, indicating that post-translational signal peptide removal occurred correctly. We could obtain over 10 mg of highly purified 22F6 light chain from a 1-liter scale culture.

### Molecular Modeling

The determined nucleotide sequence was registered in DDBJ/EMBL/GenBank^TM^ (accession number AB723624). The amino acid sequence of the light chain 22F6 was deduced from the cDNA sequence and categorized in A3/A19 of subgroup II. As mentioned above, the N-terminal amino acid sequence was MDIVM, in which M (methionine) was adducted because of the insertion of the nucleotide sequence of the restriction enzyme NcoI site.

**FIGURE 2.**
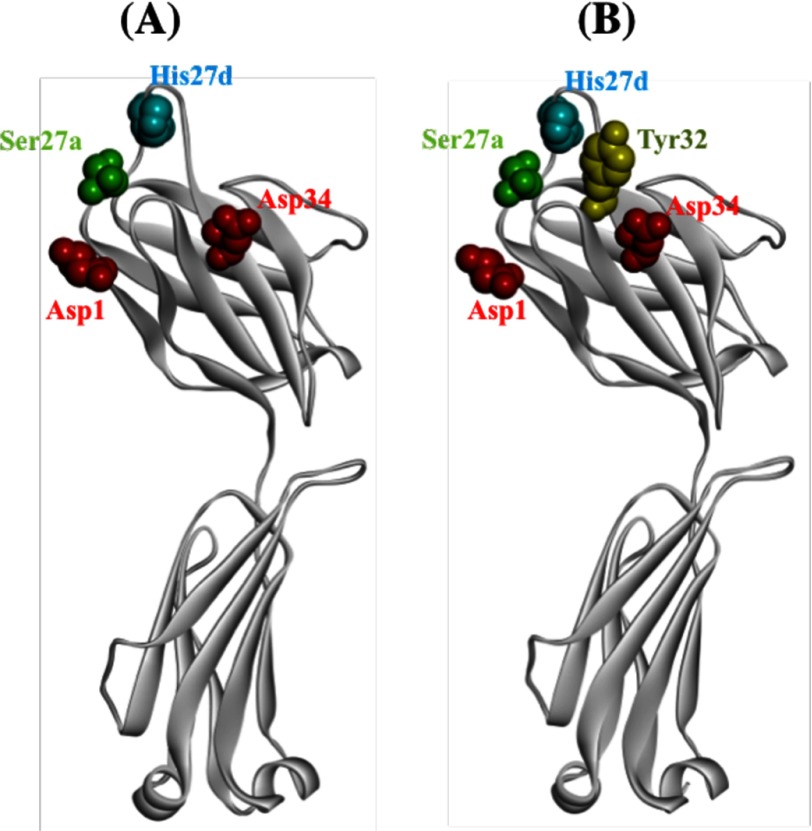
**Structure model for 22F6 light chain.** *A*, side chains having the potential to form serine protease-like triads (Asp^1^ (or Asp^34^), Ser^27a^, and His^27d^) are shown. Asp, His, and Ser are *colored red*, *blue*, and *green*, respectively. The distance between Cα of His^27d^ and Ser^27a^ is 8.26 Å, which is close to the Cα distance (8.42 Å) between them in a catalytic triad of trypsin. *B*, Tyr^32^ is visualized. The residue is situated between Asp^34^ and His^27d^ and may be concerned with the nuclease activity. Models for the light chain were visualized using WebLab ViewerLite (Accelrys Inc., San Diego, CA).

The deduced amino acid sequence was used for molecular modeling. Structure models of the light chain were constructed as shown in Fig. 2*A*. Amino acid residues Asp^1^ or Asp^34^, Ser^27a^, and His^27d^ are the candidate residues in the possible formation of a catalytic triad-like structure. The distance between His and Ser is important. In a case of the catalytic triad of trypsin, the Cα distance between His and Ser is 8.42 Å. That between His^27d^ and Ser^27a^ in 22F6 light chain is 8.26 Å. By joining Asp^1^ or Asp^34^, it might be preferable for constructing such a triad. The amino acids Asp^1^, Ser^27a^, and His^27d^ are identical to those of the *A18b* germ line gene that were found, as previously reported, to all possess amidase activity (16).

### Cleavage Assays

The functions in most catalytic antibodies reported so far were mostly investigated from the viewpoint of peptidase (1, 4, 20, 21), DNase (22–24), or RNase (25) activity. Therefore, in this study, these three kinds of catalytic features were examined as follows.

#### 

##### Hydrolysis of Synthetic Peptidyl Substrates (Amidase Activity)

**FIGURE 3.**
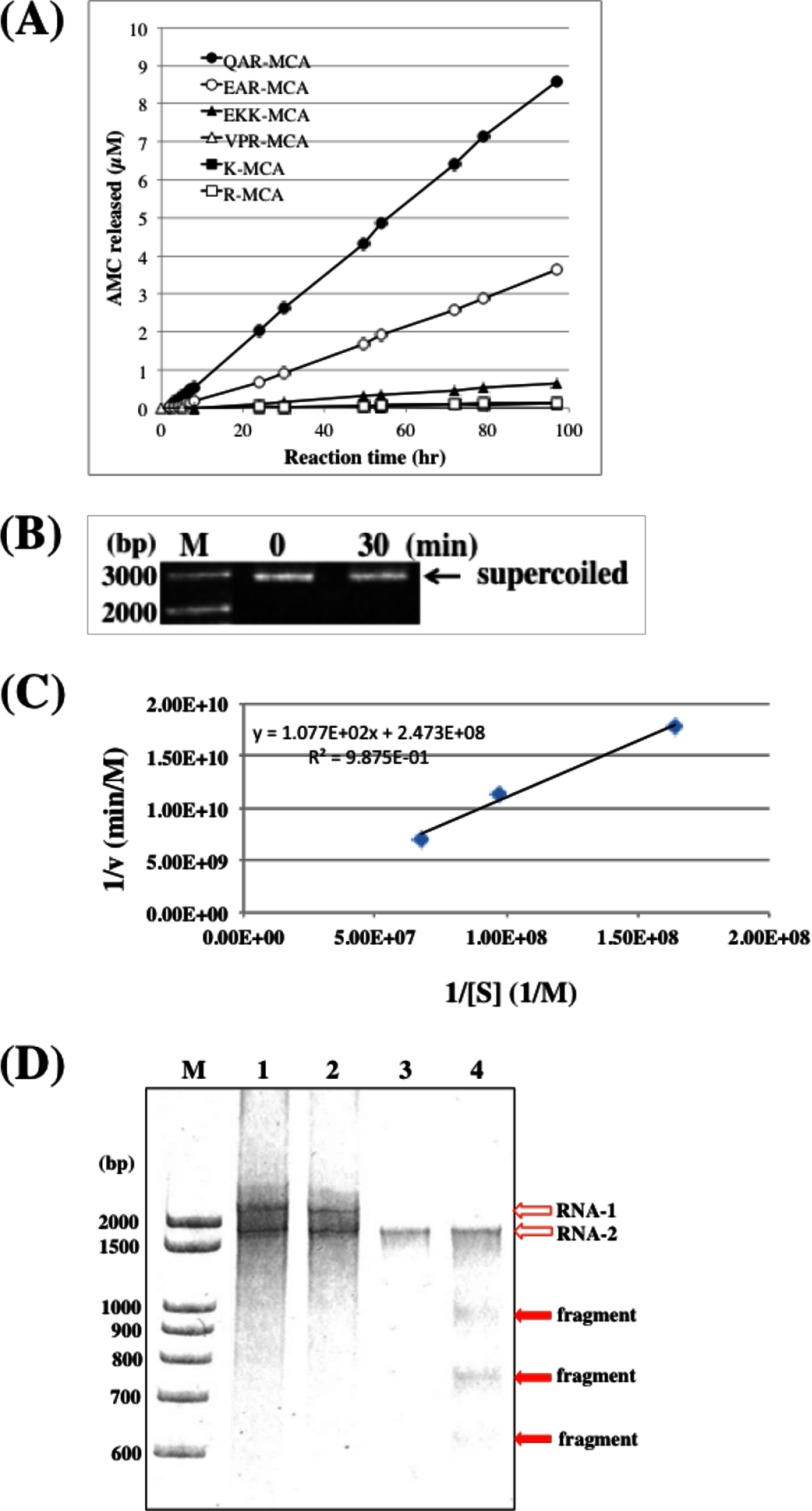
**Hydrolysis of peptide-MCA substrates and nucleic acids by 22F6 light chain.** *A*, hydrolysis of peptide-MCA substrates. The 22F6 light chain (5 μm) was mixed with an equal volume of 50 μm synthetic substrate QAR-MCA (*solid circle*), EAR-MCA (*open circle*), EKK-MCA (*solid triangle*), VPR-MCA (*open triangle*), K-MCA (*solid square*), or R-MCA (*open square*) and incubated at 37 °C. QAR-MCA and EAR-MCA were cleaved with a turnover value of 0.018 and 0.007/h, respectively. *B*, hydrolysis of a phosphodiester bond of the plasmid DNA pBR322. The 22F6 light chain inserted a nicked and a linear form of the plasmid. The nicked form as well as the linear form became clearer, and the supercoiled form became fainter at 30 min of incubation time. The hydrolysis did not occur without magnesium ion. *C*, kinetic analysis. The decrease of the supercoiled form obeyed the Michaelis-Menten equation (*k*_cat_ = 1.01 × 10^−2^/min and *K_m_* = 4.36 × 10^−7^ m). These values are comparable with those of Rodkey's catalytic antibody BV 04-01. *D*, cleavage of genome RNA from Noda virus. Noda virus possesses RNA-1 and RNA-2. In *lane 3*, RNA-1 at 3.1 kb (*open arrow*) disappeared at a concentration of 46.5 μg/ml 22F6. In *lane 4* (465 μg/ml 22F6), several fragments (*closed arrows*) generated from the parent RNA-1 could be observed at around 1,000, 760, and 630 kb. The cleavage was dependent on the concentration of 22F6. *M*, marker. *Lanes 1–4*, 0, 4.65, 46.5, and 465 μg/ml 22F6. Reaction conditions were as follows: Noda virus, 200 ng; temperature, 25 °C; period, 24 h; volume, 20 μl.

Amidase activity of 22F6 was investigated using synthetic peptidyl-4-methyl-coumaryl-7-amide (MCA) substrates. The results are shown in Fig. 3*A*. The light chain 22F6 cleaved the peptide bond between Arg and MCA of Gln-Ala-Arg (QAR)-MCA (which is mostly used as a trypsin-like substrate) with a reaction velocity of 0.09 μm/h at the concentration of 4.0 μm 22F6. For Glu-Ala-Arg (EAR)-MCA (factor Xa-like substrate), it decomposed with the reaction velocity of 0.04 μm/h. The light chain hardly cleaved Glu-Lys-Lys (EKK)-MCA (plasmin-like substrate), Arg (R)-MCA (trypsin-like substrate), Val-Pro-Arg (VPR)-MCA (thrombin-like substrate), Lys (K)-MCA (amino peptidase-like substrate), and Ala-Pro-Ala (APA)-MCA (elastase-like substrate). Gabibov and co-workers (11) obtained a catalytic antibody light chain (L12) showing a reaction velocity of 0.03 μm/h for cleavage of PFR-MCA at a concentration of 0.7 μm L12.

The tendency of the amidase activity of the light chain 22F6 to cleave synthetic substrates differs considerably from that of the other human light chains, such as chains 1, 6, 8, 10, 11, and 18, reported in a previous study (16). The 22F6 light chain seems to possess an enzymatic feature partly of trypsin and partly of factor Xa.

### Hydrolysis of Phosphodiester Bonds

#### 

##### DNase Activity

DNase activity of the light chain 22F6 was examined using pBR322 as the substrate.

As shown in Fig. 3*B,* 22F6 hydrolyzed the phosphodiester bond of the plasmid, where 0 and 30 min of incubation of 22F6 with pBR322 were presented. In the case of 30 min of incubation, the bands of the nicked and the linear forms became clearer, and the supercoiled form became fainter rather than those of 0 min of incubation, indicating a weak DNase activity. In this experiment, the hydrolysis did not take place without magnesium ion in the reaction system.

A kinetic analysis of the hydrolysis of the plasmid was performed under the following conditions: 20, 30, or 40 μg/ml plasmid, 10 μg/ml light chain 22F6, and 30 min of incubation. In the results, the reaction obeyed the Michaelis-Menten equation as shown in Fig. 3*C*, and the values of *k*_cat_ and *K_m_* were 1.01 × 10^−2^/min and 4.36 × 10^−7^ m, respectively. These values are comparable with those of Rodkey's catalytic antibody, BV 04-01, in which *k*_cat_ was 1.61 × 10^−2^/min and *K_m_* was 2.62 × 10^−8^ m (24).

##### RNase Activity

In order to investigate RNase activity, we extracted genome RNAs from influenza virus and Noda virus in accordance with past studies (17, 18). However, we failed to prepare influenza virus RNA because it was very unstable. In contrast, Noda virus RNAs (RNA-1 and RNA-2) were stable and could be successfully prepared. The RNAs were submitted to an RNase activity test using the light chain 22F6. The results are shown in Fig. 3*D*. At *lane 1*, we can see two clear bands at around 3.1 and 1.7 kb, which correspond to RNA-1 and RNA-2, respectively. The RNA-1 disappeared at a concentration of 46.5 μg/ml 22F6. At this concentration, several fragments generated from the parent RNA-1 could be seen at around 1000, 760, and 630 kb (*lane 4*), which might be consecutively hydrolyzed from the fragments at that concentration of 22F6. We could also see a smear band below the RNA-2 band. The smear band might be generated from RNA-2. The human catalytic light chain 22F6 has unique catalytic features capable of cleaving peptide bonds as well as phosphodiester bonds.

### Recognition Molecule of 22F6 in Influenza Proteins

**FIGURE 4.**
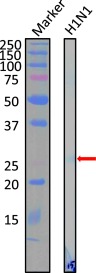
**Western blot analysis for 22F6 light chain.** Western blot of 22F6 light chain was performed against H1N1 virus proteins. A faint band was identified at around 26 kDa, which corresponds to the HA_2_ domain of the hemagglutinin molecule. The experiment used 50.9 μg/ml 22F6, which suggests that 22F6 had a lower affinity than a normal whole antibody. We could detect only the band corresponding to HA_2_, indicating that 22F6 recognized the hemagglutinin molecule.

The recognition molecule of the light chain 22F6 in proteins of influenza virus type A (H1N1) was investigated by Western blot analysis (Fig. 4), where a lysate of the virus was used. In this experiment, 50.9 μg/ml 22F6 was used, mostly employed at a concentration below several μg/ml. As already reported, the subunit of the antibody lowered the affinity to the antigen by about 1100 in the heavy chain and 11,000 in the light chain, although the recognition ability was not altered. A faint band was identified at around 26 kDa, which corresponds to the HA_2_ domain of the hemagglutinin molecule located on the surface of the virus, indicating that the light chain 22F6 could recognize the hemagglutinin molecule with a low affinity.

### In Vitro Assay; Neutralization Ability of 22F6 against Influenza Viruses

**FIGURE 5.**
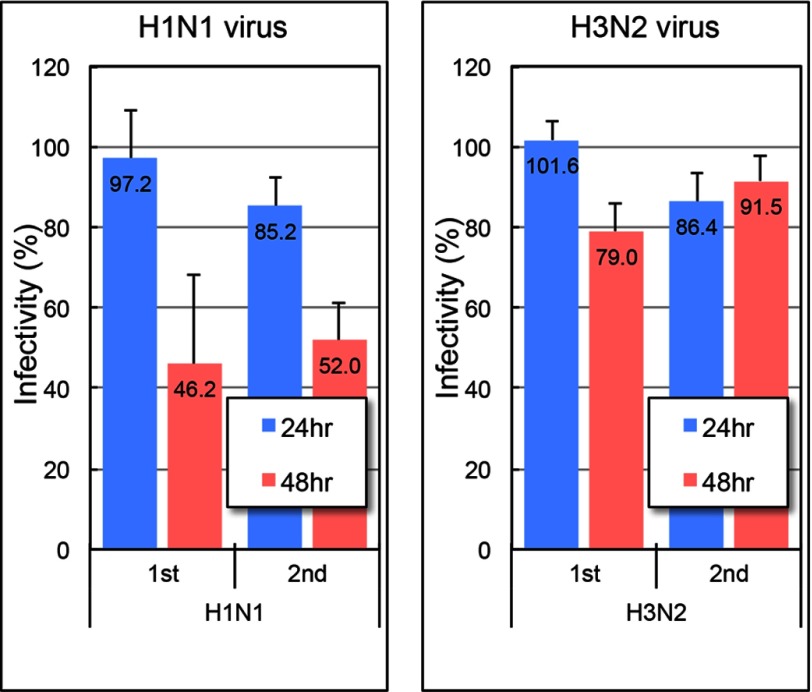
***In vitro* assays.** The human catalytic light chain 22F6 (50 μg/ml) was first incubated with 500 pfu plus 0.2 ml of influenza virus H1N1 or H3N2 for 24 or 48 h at 25 °C, and then the mixture was inoculated into the MDCK cell monolayer. The experiments were repeated twice. Although the catalytic light chain 22F6 showed little effect on the suppression of infectivity for both influenza virus H1N1 and H3N2, the catalytic light chain clearly suppressed infection of influenza virus H1N1 at 48 h of incubation. *Error bars*, S.D.

The expressed and purified light chain 22F6 was submitted to *in vitro* assays to examine the neutralization ability against both influenza viruses H1N1 (A/Hiroshima/37/2001) and H3N2 (A/Hiroshima/71/2001). In general, it requires catalytic antibodies for long term incubation (10–100 h) with its antigens because of its low turnover values. In a previous study (15), we reported that the preferable incubation temperature for a catalytic antibody with influenza virus is 20 °C for an incubation period of 48 h. Therefore, in this experiment, the human catalytic light chain 22F6 (50 μg/ml) was first incubated with 2,500 pfu/ml influenza virus H1N1 or H3N2 for 24 or 48 h at 25 °C, and then the mixture was inoculated into the MDCK cell monolayer (see “Materials and Methods”) to evaluate infectivity through the counting of the number of plaques on plates. Fig. 5 shows the effect of the human light chain 22F6 on infection of influenza virus H1N1 or H3N2 in results where *in vitro* assays were repeated twice. From the figure of the H1N1 virus (*left*) at hour 24 of incubation, the catalytic light chain 22F6 showed little effect on the suppression of infectivity of the virus. However, at hour 48 of incubation, the catalytic light chain clearly suppressed infection of the H1N1 virus down to about 50% infectivity. On the other hand, in the case of the H3N2 virus, the effect of 22F6 was low at both 24 and 48 h of incubation compared with those of H1N1 virus.

### In Vivo Assay; Survival Rate and Body Weight Changes of BALB/c Mice

**FIGURE 6.**
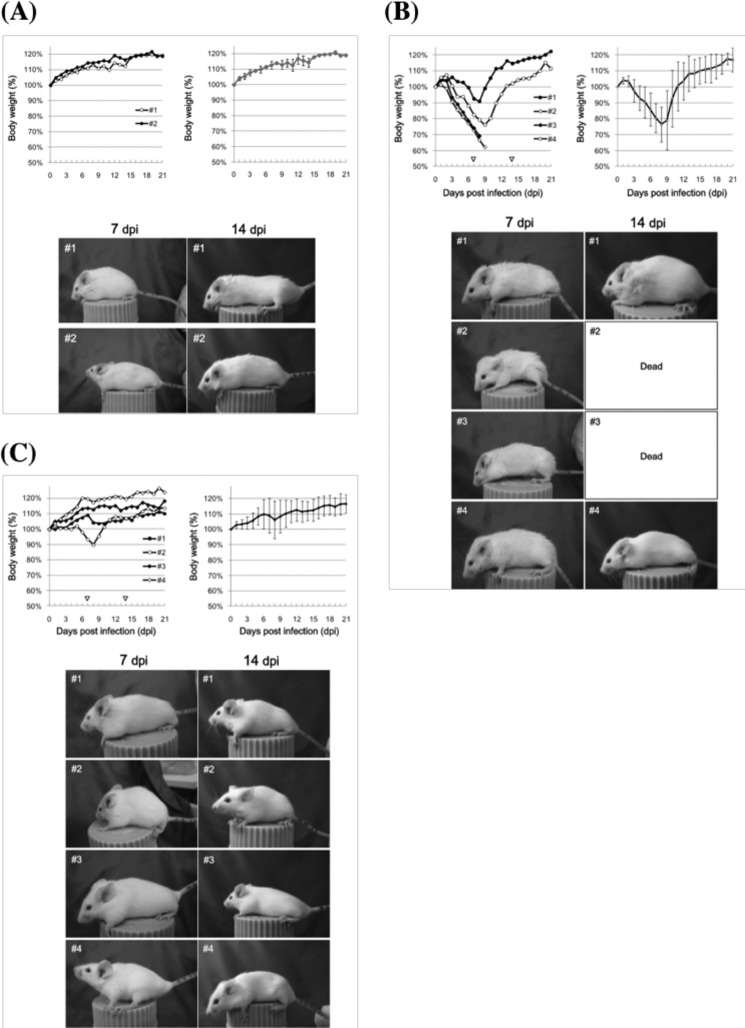
***In vivo* assays.** *A*, negative control mice (*n* = 2 mice) that received nasal administration without influenza virus (only PBS was given; mock group) were examined as shown. The time courses of the body weight are presented in the *top two graphs*. The *left graph* shows the time course of the weight for each mouse. The *right graph* shows the time course for the average body weight of two mice. The time courses of body weight were normal, as shown in the *graphs*. Photographs of the mice were taken at 7 and 14 dpi. No abnormalities were observed. *B*, positive control mice infected with intact influenza virus (PR-8 strain) were investigated. The body weight of four mice decreased until 7 dpi. All (four) mice exhibited decreases in body weight until 7 dpi. Two of the four mice died at 8 dpi (50% survival rate), and the other two mice were able to survive and recover their body weight by the final day (21 dpi). The appearance of mouse 2 and 3 was abnormal. Their bodies became smaller, and their hair bristled up. *C*, effect of 22F6 catalytic light chain. Influenza virus H1N1 (PR-8 strain) was incubated with the catalytic light chain 22F6 at 25 °C for 48 h, and then 50 μl of the mixture was given to mice (BALB/cN Sea mice, 6 weeks old, female) via nasal administration (*n* = 4 mice). One mouse decreased in body weight by 7 dpi but recovered it after 12 dpi. The other three mice increased in body weight by 21 dpi. No mice died during the assay. The human catalytic light chain 22F6 exhibited a huge effect against influenza virus infection even in the *in vivo* assay. *Error bars*, S.D.

We performed an *in vivo* assay using BALB/c mice and influenza virus H1N1 (PR-8 strain). The time course of negative control mice (*n* = 2 mice), which received nasal inoculation without the influenza virus (50 μl of PBS was given; mock group), is shown in Fig. 6*A*. The time course of the body weight of each mouse was normal, as shown in the graphs in the Fig. 6*A*. Photos of the mice were taken at 7 and 14 dpi. Their appearance was also normal. No mice died in this negative control experiment.

In Fig. 6*B*, an investigation of positive control mice infected with intact influenza virus (50 μl of PBS containing 1,000 pfu/50 μl; positive control group) indicated that the body weight of the four mice decreased until 7–9 dpi. All (four) mice exhibited decreases in body weight, and two of the four mice died at 8 dpi (50% survival rate); the other two mice were able to survive and recovered their body weight by the final day (21 dpi).

The influenza virus was incubated with the catalytic light chain 22F6 at 25 °C for 48 h, which was similar to the treatment in the *in vitro* experiment. Then 50 μl of the mixture was given to mice via nasal inoculation (*n* = 4 mice). The body weight changes and survival rate of the mice were examined as shown in Fig. 6*C*. One mouse decreased in body weight by 7 dpi but recovered it after 12 dpi. The other three mice gradually increased in body weight by 21 dpi. The appearance of the four mice, judging from the photos taken at 7 and 14 dpi, reveals that the mice were normal. Note that no mice died even at 21 dpi in this experiment. The catalytic light chain 22F6 obviously improved the survival rate as well as the loss of body weight.

**FIGURE 7.**
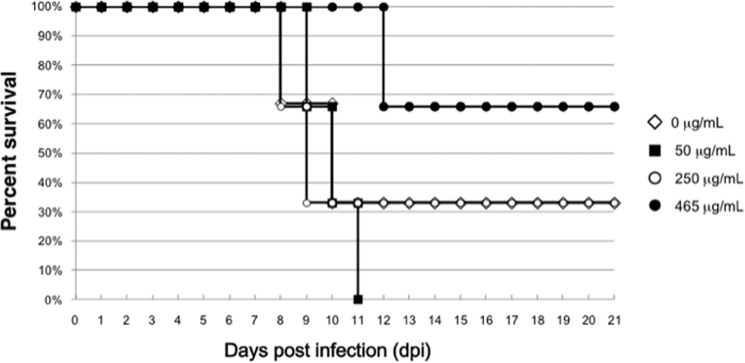
**Dose dependence.** The dose dependence of 22F6 was investigated. In this experiment, the virus at 2,000 pfu/50 μl was employed. Administration of 50 μg/ml 22F6 did not show an effect (0% survival), but that of 465 μg/ml 22F6 substantially improved the survival rate (65%) of the mice.

Dose dependence of 22F6 was also investigated, as shown in Fig. 7. In the experiment, the virus in nasal inoculation was increased from 1,000 to 2,000 pfu/50 μl in order to clearly ascertain the differences. Incubation of 50 μg/ml 22F6 with influenza virus H1N1 did not show an effect (0% survival), but that of 465 μg/ml 22F6 substantially improved the survival rate (65%) of the mice, indicating a clear dose dependence.

**FIGURE 8.**
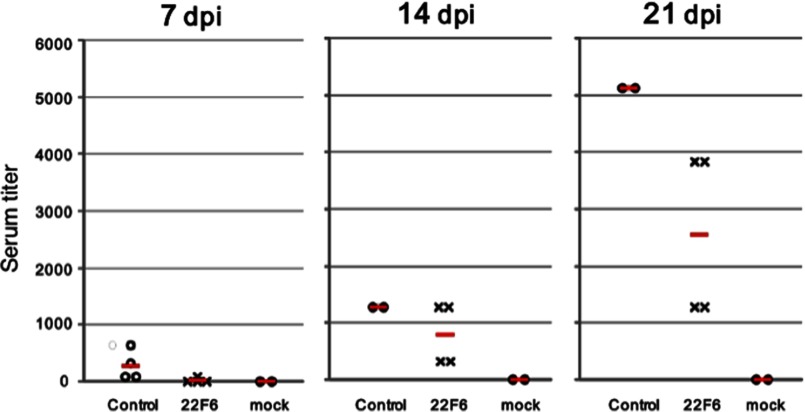
**Serum titer.** The antibody titer against influenza virus in the mice serum tested in the above *in vivo* assay was examined. The titer of control mice infected with intact virus gradually increased, depending upon the time course measured. At 21 dpi, the titer reached 5,000-fold, whereas mock mice inoculated with only PBS did not show any response. On the other hand, the titer of the mice infected with a solution including intact virus and 22F6 catalytic light chain was 2,500-fold at 21 dpi, which was significantly lower than that of the positive control mice. This means that some part of the antigenicity of the influenza virus was lost by contact with the catalytic light chain 22F6.

From an immunological point of view, we examined the antibody titer against influenza virus in the mice serum tested in the above *in vivo* assay (Fig. 6, *A–C*). In Fig. 8, the antibody titer against the influenza virus is indicated on the *vertical axis* at 7, 14, and 21 dpi. The titer of control mice infected with intact virus gradually increased, depending upon the time course measured. At 21 dpi, the titer reached to 5,000-fold (*vertical axis*). On the other hand, that of the mice infected with a solution including intact virus and 22F6 catalytic light chain was 2,500-fold at 21 dpi, which was significantly lower than that of the control mice. This means that some part of the antigenicity of the influenza virus was lost by contact with the catalytic light chain 22F6. Mock mice inoculated with only PBS did not show any response. These data indicate that the human light chain 22F6 possesses the ability to suppress infection of influenza virus H1N1 (PR-8 strain) *in vivo*.

### Investigation of Toxicity of Human Light Chain

Acute toxic features of the human light chain were investigated.

#### 

##### Single-dose Inoculation

**FIGURE 9.**
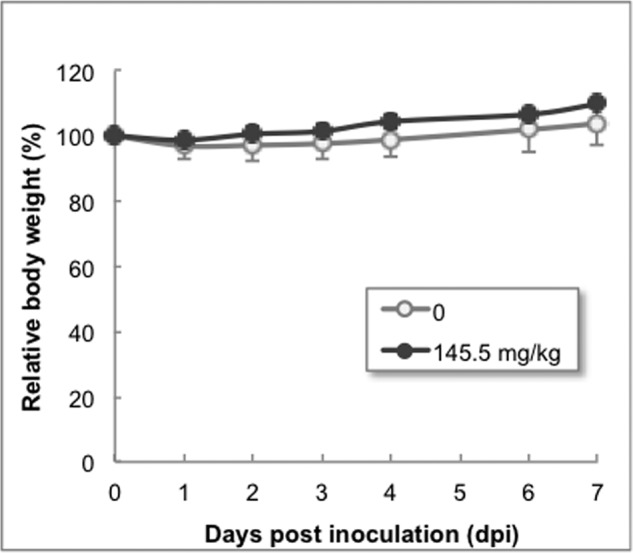
**Acute toxicity tests.** The time courses of the body weight of mice administered 145.5 mg/kg at single dose are shown. No significant differences between the administered (●) and control (○) mice were observed. *Error bars*, S.D.

The oral and intraperitoneal inoculations of the mice (*n* = 3) consisted of 33.2 and 49.8 g/kg/day, respectively. No abnormal findings were observed in these two experiments. The intravenous inoculation (inoculation volume = 15 ml/kg/day) was made up of, in detail, injections at several concentrations, including 8.3, 24.9, 49.8 (1.66 mg/ml), and 145.1 (9.67 mg/ml) mg/kg/day. For all of the single-dose inoculations, nothing particularly noteworthy was observed at all from the viewpoints of body weight, death rate (0%), and dissection of the cranial, intrathoracic, and abdominal cavities and lymph nodes of the mice in addition to their appearance. The time course of the body weight of mice intravenously inoculated with 145.5 mg/kg is shown in Fig. 9, where no significant differences were observed between the inoculated and control mice (*n* = 3 mice for each group).

##### Multiple-dose Inoculation

Multiple doses of 8.3 mg/kg were given to mice (*n* = 3) by intravenous inoculation of 8.3 mg/kg (inoculation volume = 5 ml/kg, 1.66 mg/ml) every 7 days. No remarkable appearance or occurrences were observed in body weight, food and water intake, and dissection. The weight of each organ was measured, and no unusual findings were observed. The brain, thymus, heart, lung, liver, kidney, adrenal gland, and spleen were compared between non-inoculated and inoculated mice. In observations from this dissection, we could not detect any abnormal occurrences (data not shown).

## DISCUSSION

In the last 2 decades, many functional catalytic antibody light chains have been reported worldwide by many researchers (26–30). Past studies point out that the active site of a catalytic antibody resides in the variable region of the antibody and not in the constant region (1, 20, 31). Catalytic antibody light chains, such as an anti-vasoactive intestinal peptide antibody light chain (1, 26), i41SL1–2 light chain (cleaving small peptides) (32), ECL2B-2 light chain (cleaving CCR-5 peptide) (13), and UA15 light chain (cleaving urease of *H. pylori*) (14) have three identical amino acids (Asp^1^, Ser^27a^, and His^93^). Ueda and co-workers (33) revealed the important role of the residues of the three amino acids in a light chain exhibiting amidase activity by genetically mutating the residues, using their mouse antibody light chain. Uda and Hifumi (20) have already proposed, using molecular modeling for 128 mouse κ-type germ line genes, that the catalytic sites composed of Asp, Ser, and His in mouse light chains are mostly encoded in certain particular germ line genes, such as *bd2*, *bb1*, *cr1*, *bl1*, *cs1*, and *bj2*. Based on the above concept applied to human cases, Vκ germ line genes, such as *A3/A19*, *A5*, *A17*, *A18*, and *A23* of subgroup II of human light chains, were considered to correspond to the mouse germ line genes. In our previous work (16), catalytic light chain 18, which belongs to the *A18* germ line gene, showed an ability to suppress the infection of rabies virus both *in vitro* and *in vivo*. In this study, we were able to successfully obtain the highly purified catalytic light chain 22F6, possessing interesting catalytic features, which was prepared from human B-lymphocytes. Regarding amidase activity, the light chain 22F6 cleaved the synthetic peptidyl substrates of QAR-MCA and EAR-MCA with a reaction rate (*k*_cat_ value) comparable with that of light chain 18 (12), but it did not cleave VPR-MCA and EAR-MCA at all, which have very different characteristics from light chain 18 reported previously (16). In addition, 22F6 possesses an ability to insert a nick into pBR322 and convert to a linear form through successive hydrolysis of phosphodiester bonds.

Kinetic parameters reported for catalytic antibodies showing amidase, peptidase, DNase, and RNase activity are summarized in Table 1, in which several forms of catalytic antibodies, such as light chain alone, scFv, IgG, and pIgG, are listed. Regardless of either synthetic peptides or nucleic acids as the substrate, most catalytic antibodies have a *K_m_* value in the range of 10^−3^ to 10^−5^ m and a *k*_cat_ value from 10^−3^ to 1.0 min^−1^. 22F6 in this study showed a cleaving velocity (*V*) similar to that of the L12 light chain. Regarding DNase or RNase activity, 22F6 showed DNase activity comparable with that reported by Rodkey *et al.* (24) (BV 04-01 antibody). In both Rodkey's and our cases, magnesium ion was necessary. Because 22F6 cleaved Noda virus RNA (Fig. 3*D*), it is characteristic that 22F6 showed both amidase and nuclease activity.

**TABLE 1:** **Kinetic parameters for catalytic antibodies showing amidase (peptidase) and/or nuclease activity**

Name	Form	Substrate	*K_m_*	*k*_cat_	Reference/Source	Remarks
			*m*	*min*^−*1*^		
HIR (BJP)	L	Chromozyme TRY	1.5 × 10^−4^	6.2	Ref. 34	
RHY	L	IEGR-MCA	1.03 × 10^−4^	2.62 × 10^−2^	Ref. 12	
E6	scFv	LAEEEV-MCA	5.3 × 10^−5^	1.1 × 10^−3^	Ref. 35	
L12	L	PFR-MCA	5.3 × 10^−5^	1.55 × 10^−3^	Ref. 35	*V* = 0.03 μm/h (0.7 μm L) turnover = 0.04/h
22F6	L	QAR-MCA	Not measured	Not measured	This study	*V* = 0.09 μm/h (4 μm L) turnover = 0.02/h
6B8-E12	IgG	AAPFpNa	1.4 × 10^−3^	1.5	Ref. 36	
6B8-E12	scFv	Leu-MCA	5 × 10^−5^	0.52	Ref. 36	
YZ17	Fv	EAR-MCA	1.01 × 10^−5^	0.5	Ref. 37	
Hybrid IgG	IgGn	EAR-MCA	1.546 × 10^−4^	1.77 × 10^−2^	Ref. 38	
BV 04-01	scFv	plasmid DNA	2.62 × 10^−8^	1.61 × 10^−2^	Ref. 39	
Human pIgG(2)	pIgG	scDNA	7.6 × 10^−5^	1.5 × 10^−3^	Ref. 9	
Rabbit pAB	pAB	(pC)_9_	9 × 10^−4^	1.7	Ref. 10	
Rabbit pAB	pAB	(pU)_9_	2.9 × 10^−3^	1.0	Ref. 10	
22F6	L	pBR322	4.36 × 10^−7^	1.02 × 10^−2^	This study	

Rodkey's catalytic antibody (BV 04-01) forms a catalytic site composed of His^27d^ and Tyr^32^ in a light chain. In the case of 22F6 light chain, the two amino acids are present at positions identical to those of Rodkey's catalytic antibody. In Fig. 2*B*, Tyr^32^ situates between His^27d^ and Asp^34^ in CDR-1 of 22F6. His^27d^ may be involved in the formation of both the nuclease and amidase active sites. Thus, it is conceivable that the DNA and RNA cleaving site (His^27d^ and Tyr^32^) may co-exist with the active site as amidase (Asp^1^ (or Asp^34^), Ser^27a^, His^27d^) in 22F6. Namely, two different active sites may be present in one light chain, although it is reported that the heavy chain is concerned with the binding to DNA (40).

There have been many reports with respect to the structure of catalytic site in antibody or its subunits. Kolesnikov *et al.* (41) found a dyad structure, where the anti-idiotypic mAb (9A8) displays esterolytic activity. Both the His^35^ and Ser^99^ residues reside in the heavy chain and act as the catalytic site. The dyad structure is not a special case. For instance, a signal peptidase forms the dyad catalytic site composed of amino acid residues (Ser^90^–Lys^145^) to cleave the peptide bond of a signal peptide (42). Recently, Smirnov *et al.* (11) proposed a unique catalytic antibody named the “reactibody,” which was prepared by employing an innovative idea and technique. The A17 could cleave paraoxon, where tyrosine positioned at 37 in the light chain was mainly concerned with the enzymatic function). Anyhow, it seems that some amino acids in CDR-1 in light chain are often concerned with the formation of catalytic site.

Note that precise analysis of a three-dimensional structure of a catalytic light chain is not the primary purpose of this work. Rather, the purpose is to find potent light chain candidates and then investigate their biological features.

In an *in vitro* assay, the catalytic light chain 22F6 was able to substantially suppress infection of influenza virus H1N1. Incubation was more effective after 48 h than 24 h. This means that 22F6 took the time to perform this function. On the other hand, the monoclonal antibody C179 (which is a well known neutralizing antibody against H1N1) did not show such dependence on incubation time. The monoclonal antibody completed binding with the antigen within a short time. In contrast, a huge dependence on incubation time was observed in the case of the catalytic light chain 22F6, suggesting that 22F6 was working as an enzyme in addition to binding to the antigen, followed by degradation of influenza virus protein or RNA.

Note that the catalytic light chain 22F6 obviously improved the survival rate as well as the body weight of mice when the catalytic light chain was coinfected with influenza virus. Fig. 8 presents the important finding that the catalytic light chain must make the antigenicity of the H1N1 virus decrease through a catalytic feature of the light chains. The relationship between such catalytic features and the ability to suppress influenza virus infection is still unclear. However, based on the above findings of enzymatic activity, binding to the hemagglutinin molecule, decrease of the antigenicity of influenza virus, and hydrolysis of Noda virus RNA1, there must be two possibilities for 22F6 to possess anti-virus activity. One is the degradation of the hemagglutinin molecule present on the surface membrane of the virus. Another is the decomposition of genome RNA of the virus, in which 22F6 penetrates the virus membrane and enters into the virus. We assayed the degradation of the hemagglutinin molecule by 22F6. However, the degraded fragments of the hemagglutinin molecule were hardly observed. In contrast, the genome RNA (RNA-1) of Noda virus was clearly decomposed by 22F6, suggesting that 22F6 could possess the ability to cleave RNA of influenza virus. Actually, as the loss of infectivity of the influenza virus took place, it is plausible that 22F6 could degrade the RNA and reduce the antigenicity. This assumption will be investigated in near future. Anyhow, it is characteristic that 22F6 human catalytic light chain possesses the strong suppression activity for the infection of influenza virus *in vivo* as well as *in vitro*.

Through the above data and discussion, we should note the potency of our immune system once again. The light chain, such as 22F6, can be produced in our bodies. It became well known that the free light chains released in our bodies play important roles for such diseases of AIDS, allergies, etc. (43). In the case of this study, it is plausible that free antibody light chains produced and released into our bodies are advantageous to our health. In conclusion, because we could not observe any problems with antibody light chains in acute toxicity tests, the effective catalytic light chain holds huge potential as a new drug for patient therapies in the future.

## Supplementary Material

Supplemental Data

## Footnotes

2 The abbreviations used are:

MDCK: Madin-Darby canine kidney
dpi: days postinoculation
MCA: 4-methyl-coumaryl-7-amide
pAb: polyclonal antibody.
