# Supplementary material for: Biochemical Features of a Catalytic Antibody Light Chain, 22F6, Prepared from Human Lymphocytes
Source: J Biol Chem. 2013 May 15;288(27):19558–68. doi: 10.1074/jbc.M113.454579 (PMC3707657; doi:10.1074/jbc.M113.454579)
Supplement: Supplemental Data [file supp_288_27_19558__index.html]

Biochemical features of a catalytic antibody light chain, 22F6, prepared from human lymphocytes — Biochemical Features of a Catalytic Antibody Light Chain, 22F6, Prepared from Human Lymphocytes — Biological Features of a Human Catalytic L Chain 22F6 — Supplemental Data 

# Biochemical Features of a Catalytic Antibody Light Chain, 22F6, Prepared from Human Lymphocytes

## Supplemental Data

**Files in this Data Supplement:**

- JBC-R1-final-1BW-1 (.pdf, 261 KB) - SDS-PAGE, in gel assay and image analysis for 22F6 light chain. (Supplemental date to answer #2 reviewer's comments.)
