# Supplementary material for: Biochemical Features of a Catalytic Antibody Light Chain, 22F6, Prepared from Human Lymphocytes
Source: J Biol Chem. 2013 May 15;288(27):19558–68. doi: 10.1074/jbc.M113.454579 (PMC3707657; doi:10.1074/jbc.M113.454579)
Supplement: Supplemental Data [file supp_M113.454579_jbc.M113.454579-1.pdf]

(Supplement) Figure S1

In CBB staining under the non-reduced condition (left figure) in Fig. S1, we observed the bands of the 22F6 light chain at 51-kDa as the dimer and 26-kDa as the monomer. No band was detected at 34-kDa in CBB staining, which was the DNase I used in this experiment.

The corresponding in-gel assay, which was performed in accordance with the reference of Nevinsky (9), was presented at the right hand side of the CBB staining. We could observe a strong band corresponding to the dimer and a faint band corresponding to the monomer. This means that the dimer possesses much stronger cleavage ability for DNA than the monomer. A band appeared at the middle position (about 34-kDa), which is very close to that of DNase I, between the dimer and monomer. This faint band may be generated by the small contamination. In addition, a faint peak detected at the position between the dimer and DNA I is considered to be a different or fragmented form of 22F6, because we can see the band at the same position in CBB staining.

In order to estimate the percentage of contribution of DNase, we performed a quantitative analysis for the gel of 3.50μg/lane of 22F6 using an image analyzer (Tyhoon-9400, Amersham Bioscience). The result was presented at the right hand side of Fig. S1. The area of the band (peak 2; corresponding to the position of the DNase) at 34-kDa became about 10% in the total area. Namely, even if the small contamination is present in this experiment, it was at most around 10%. Thus, our main stream in this study is not so influenced even if the contamination took place. (Of course, no contamination might be occurred).

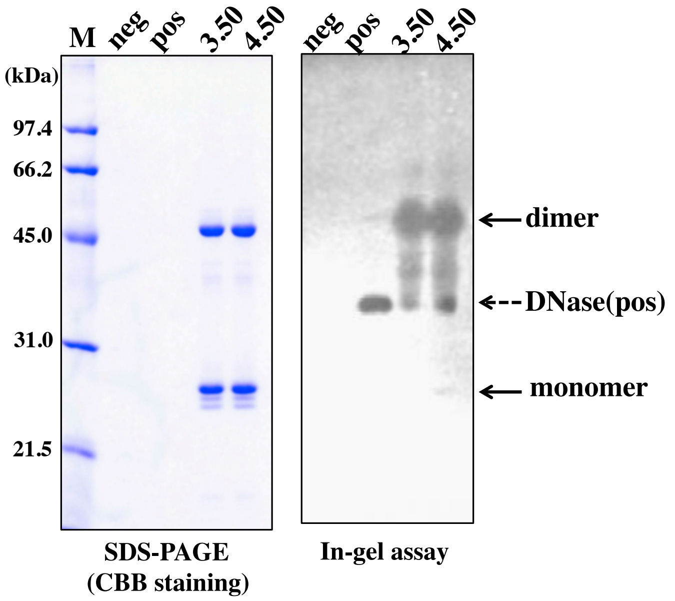

Positive: DNase I, 0.05 U/lane

Negative: 0.1%SDS + 20mM Tris-HCl

22F6: 3.50 μg/lane 22F6: 4.50 μg/lane

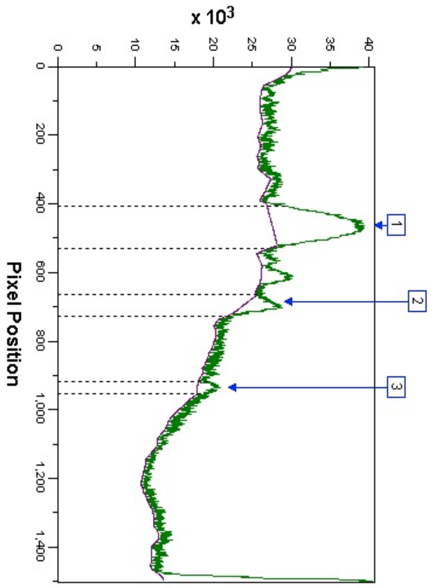

"1" is the dimer and "3" monomer. "2" corresponds the position of DNase I. The area is about 10% of total area. (22F6; 3.50 μg/lane)

Fig. S1

SDS-PAGE, in gel assay and image analysis for 22F6 light chain.
